# Development and Investigation of a Polyarylene Ether Nitrile Coating Material as Corrosion Protection for Metal Substrates

**DOI:** 10.3390/ma19091837

**Published:** 2026-04-29

**Authors:** Yunqing Xia, Shaomu Wen, Hongfa Huang, Jin Yan, Hongjie Li, Lincai Peng

**Affiliations:** 1 Research Institute of Natural Gas Technology, Southwest Oil & Gasfield Company, Chengdu 610213, Chinalincai.peng@foxmail.com (L.P.); 2 Southwest Oil & Gasfield Company, Chengdu 610051, China

**Keywords:** polyarylene ether nitrile, mechanical properties, corrosion resistance, epoxy

## Abstract

**Highlights:**

- Polyarylene ether nitrile (PEN) with bisphenol A structure was first employed as a candidate coating in downhole tubing within the oil and gas field, and its relevant comprehensive properties were validated.
- PEN coatings provide better corrosion resistance, thermal stability and mechanical properties than commercially established high-temperature-resistant epoxy coating.
- Polyarylene ether nitrile material could be employed as an alternative candidate to epoxy coating and used in protecting metallic substrates in the oil industry against corrosion.

**Abstract:**

In this research, a novel polyarylene ether nitrile (PEN) coating material was fabricated through a facile stepwise polymerization method, which provides metallic substrates used in the oil industry with remarkable corrosion protection performance. A variety of characterization techniques were employed to evaluate the comprehensive properties of the PEN coating materials against a commercially established high-temperature-resistant epoxy coating. Based on the TGA curves, the PEN3 coating exhibited a T_5%_ value of 521 °C, which was 44.72% higher than that of the epoxy coating. According to the tensile experiment, the PEN coatings demonstrated improved mechanical performance, achieving tensile strength and breaking elongation values of 89.37 MPa and 7.14% (PEN3), respectively, while the epoxy achieved values of 18.67 MPa and 0.32%, respectively. EIS tests revealed that all the PEN coatings exhibited superior corrosion resistance compared to the epoxy coating. Among them, the PEN3 coating remained intact without failure and showed the highest impedance value (5.665 × 10^7^ Ω·cm^2^), which was two orders of magnitude higher than epoxy. Our research confirmed that the PEN coating material provided enhanced corrosion resistance, thermal stability and mechanical properties, positioning it as an alternative option to replace epoxy coating in prolonging the service life of steel piping in oil field applications.

## 1. Introduction

The integrity of metal oil tubing is critically important for the safe and efficient exploitation of oil and gas resources [1,2]. However, internal corrosion-induced perforation and degradation of metal tubing, which is driven by complex service conditions (such as high temperature, high pressure and highly corrosive media), persist as challenges in pipeline protection [3,4,5]. Currently, epoxy anti-corrosion coatings with high-temperature resistance are widely employed for internal corrosion protection within metal oil tubing due to their remarkable overall performance and high cost-effectiveness [6,7,8]. These coatings function as a dense organic barrier that prevents direct contact between corrosive media and metallic substrate, thereby effectively extending the service life of metal pipelines [9,10]. Unfortunately, thermosetting pipeline coatings typified by bisphenol A epoxy resin demonstrates an excessively high crosslinking density, restricting rapid stress dissipation and resulting in high brittleness [11,12,13]. Consequently, stress concentration in epoxy coating leads to cracking and failure under high-temperature and high-pressure operating conditions, which allows corrosive agents to rapidly penetrate the damaged areas and erode the underlying metal surface. Therefore, the exploration of alternative corrosion protection coatings and strategies to ameliorate epoxy resins’ brittleness has emerged as a critical research priority.

In recent years, thermoplastic resins have not only been widely used as excellent modifiers to enhance the toughness of epoxy resins, they also have been employed as high-performance protective coatings in the field of metal substrate protection [14,15,16]. Polyarylene ether nitrile is a high-performance thermoplastic characterized by a backbone consisting of rigid aromatic rings and flexible ether linkages [17]. Owing to this distinctive molecular architecture, polyarylene ether nitrile exhibits a desirable combination of high mechanical strength, thermal resistance, and excellent flexibility [18,19,20]. Furthermore, the presence of strongly polar cyano groups along the polymer chain induces significant intermolecular interactions, endowing polyarylene ether nitrile with outstanding overall capacities compared to other thermoplastics commonly used for epoxy toughening, such as polyetheretherketone (PEEK) [21,22], polyethersulfone (PES) [23] and polyurethane (PU) [24]. As a result, polyarylene ether nitrile has accumulated considerable applications across various fields. Notably, in addition to its high thermal stability and outstanding mechanical properties, polyarylene ether nitrile also demonstrates excellent corrosion resistance, barrier performance, chemical solvent resistance, and electrical insulation [25,26,27]. Moreover, the strong polar cyano side groups in polyarylene ether nitrile enable favorable interactions with metallic surfaces, resulting in good adhesion. These attributes satisfy the fundamental performance requirements for anticorrosive pipeline coatings, further supporting the feasibility of applying polyarylene ether nitrile independently as a high-temperature-resistant protective coating for metal pipelines.

Currently, little research has focused on the application of polyarylene ether nitrile as an anticorrosive coating for metal pipelines. Moreover, the majority of studies employed polyarylene ether nitrile as an efficient modifier to ameliorate epoxy coating’s thermal and mechanical capacities within the field of corrosion protection. Thus, to advance the scientific exploration of polyarylene ether nitrile in the field of corrosion protection, the present study systematically evaluated its key properties and attributes as an alternative anticorrosive coating material for metallic substrates to a current high-temperature-resistant epoxy coating used in downhole tubing applications. This study aims to verify polyarylene ether nitrile’s feasibility for such applications, thereby establishing a foundational basis for subsequent further investigation.

## 2. Materials and Methods

### 2.1. Materials and Reagents

The chemical reagents were applied without further purification in this experiment. Detailed information of the reagents is displayed in Table 1.

### 2.2. Synthesis of PEN Materials

**Figure 1:**
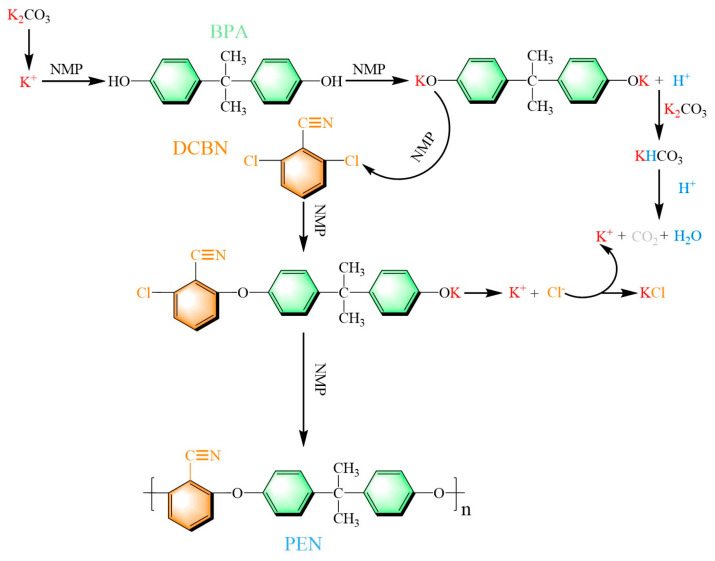
Synthesis route for the preparation of PEN.

PEN was synthesized via a facile nucleophilic substitution polymerization, which consisted of DCBN and BPA. This particular synthesis route is illustrated in Figure 1. Firstly, equimolar amounts of DCBN, BPA, and K_2_CO_3_ were transferred to a three-necked flask, with NMP and toluene assisting as solvents. The mixed solvent was heated up to 140 °C and maintained at a stirring rate of 500 rpm for the dehydration process. During this reaction stage, K^+^ generated from K_2_CO_3_ in the mixture reacted with BPA to form BPA dipotassium salt and H^+^. Meanwhile, H^+^ conducted a side reaction process with HCO_3_^−^ (formed from K_2_CO_3_ and H^+^), through which CO_2_ and H_2_O were produced. After azeotropic dehydration at 140 °C, residual water was removed using a condenser and a water separator. The temperature was then increased to 200 °C, and the reaction mixture was stirred for several hours to acquire PEN with varying molecular weights, designated as PEN1, PEN2, and PEN3 (the polymerization times required for different molecular weights are provided in Table 2). Upon polymerization, the crude PEN product was diluted with an appropriate amount of NMP to reach a suitable viscosity and subsequently precipitated into deionized water containing 5 wt% HCl. The obtained mixture was washed 3 times to remove residual unreacted K_2_CO_3_, followed by repeated washing with boiling water until it reached neutrality. The purified product was then pulverized into a fine powder using a grinder. After the drying process, the PEN powder was subjected to reflux purification in ethanol to eliminate unreacted low-molecular-weight residues. The product was washed again with ethanol and dried in an oven at 100 °C for 24 h to obtain the final PEN product. The yields of PEN1, PEN2, and PEN3 were 96.2%, 98.0%, and 97.8%, respectively.

### 2.3. Fabrication of Coating Materials

#### 2.3.1. Fabrication of Coating Films

PEN samples with different molecular weights (1 g each) were dissolved in 10 mL of CYC solvent. After thorough stirring to achieve a homogeneous and clear solution, the PEN solution was cast onto a glass plate measuring 12 cm × 12 cm. The plate was then placed in a forced-air oven and subjected to the following heating program, with a ramp rate of 2 °C/min: maintained at 100 °C and 120 °C for 1 h each, at 140 °C and 160 °C for 2 h each, and finally at 200 °C for 3 h. The resulting PEN coating films exhibited an average thickness of 65 ± 5 µm. For the purpose of conducting a comparative experiment, E-51 epoxy resin (1.5 g) and curing agent (0.15 g) were also dissolved in 10 mL of CYC solvent to fabricate epoxy coating films (65 ± 5 µm), following an identical fabrication procedure to that of the PEN coating films.

#### 2.3.2. Fabrication of Coating Samples

The P110 steel sheets (50 mm × 10 mm × 3 mm) intended for electrode coating preparation, the tinplate sheets (150 mm × 100 mm × 0.5 mm) for salt spray tests and the iron sheets (50 mm × 50 mm × 5 mm) for adhesion tests were first subjected to surface roughening via a sandblasting machine to achieve Sa2.5 grade. Subsequently, the substrate surfaces were cleaned with ethanol and dried using a hairdryer set to cool air.

Masses of 1.5 g of PEN with varying molecular weights were each dissolved in 10 mL of CYC until a clear solution was obtained. The resulting solution was then moved to a vacuum oven for degassing for 30 min. Following the degassing process, the PEN solution was transferred to a spray gun and applied onto the pre-treated metal substrates via high-pressure air spraying. The spraying procedure varied depending on the sample type: electrode coating samples were sprayed four times, salt spray samples twice, and adhesion test samples five times. Notably, during the multi-layer spray coating process, each layer was permitted to reach surface dryness (maintained at 80 °C for a proper time) prior to application of the next layer. This sequential approach was consistently employed to ensure proper interlayer adhesion and to achieve the desired coating thickness without compromising coating quality. After spraying, the coated samples were placed in a forced-air drying oven and subjected to a stepwise curing process. The temperature was increased within a controlled rate of 2 °C/min, with the samples held at 80 °C, 100 °C, and 120 °C for 1 h each, subsequently at 140 °C and 160 °C for 2 h each, and finally at 200 °C for 3 h to acquire the finished coating samples. The resulting coating thicknesses were controlled to 80 ± 5 µm for the P110 steel sheets used in electrochemical experiments, 40 ± 5 µm for the tinplate sheets used in salt spray tests, and 120 ± 5 µm for the sheets used in adhesion tests. For comparison purposes, 5 g of E-51 epoxy resin and 0.5 g of curing agent were also dissolved in 10 mL of CYC solvent to fabricate the epoxy coating, following same heating procedure used for the PEN coating. It is worth noting that electrode coating samples were sprayed twice (40 ± 5 µm), salt spray samples once (40 ± 5 µm), and adhesion test samples three times (120 ± 5 µm).

### 2.4. Characterization

#### 2.4.1. Characterization of Fabricated PEN Materials

The molecular weights of the synthesized PEN samples were determined using gel permeation chromatography (Agilent, Waters 1515, Santa Clara, CA, USA), with tetrahydrofuran as the mobile phase. The chemical structures of the PEN samples with different molecular weights were characterized by proton nuclear magnetic resonance spectroscopy (Bruker, Bruker Avance III 400 M, Billerica, MA, USA). Deuterated dimethyl sulfoxide was used as the solvent, and the measurements were performed at 600 MHz. The structural states of the PEN samples with different molecular weights were analyzed by X-ray diffraction (Rigaku, Ultima IV, Tokyo, Japan) via Cu Kα radiation (λ = 1.5418 Å) generated at 40 kV and 40 mA. Measurements were performed across a 2θ interval of 5–50° using a step increment of 0.02°.

#### 2.4.2. General Characterization of Ep and PEN Coatings

Thermal stabilities of PEN coatings with different molecular weights and the epoxy coating were determined using a thermogravimetric analyzer (TA, Q50, New Castle, DE, USA) under a nitrogen atmosphere. The samples were heated from 50 to 800 °C at a heating rate of 20 °C/min. The tensile properties of the PEN and EP coatings were assessed using a universal testing machine (SANS (Shenzhen) Experimental Equipment Co., Ltd., CMT6104, Shenzhen, China) in accordance with the GB/T 1040.3-2006 standard [28], at a crosshead speed of 5 mm/min. Each sample group was tested in triplicate to minimize errors. The toughness of the coated samples was evaluated with a paint film impact tester (Shanghai Meiyu Testing Instrument Co., Ltd., QCJ-120, Shenzhen, China), following the GB/T 1732 standard [29]. Adhesion strength of the PEN and epoxy coatings with different molecular weights was assessed using a pull-off adhesion tester (Xinghuo Technology Development Co., Ltd., XH-M, Shenzhen, China) through the GB/T 5210-2006 [30]. The average thickness of the coatings was 120 ± 5 µm. Microstructural analysis of the cross sections of PEN and epoxy resin specimens was performed using a scanning electron microscope (Thermo Scientific, Apreo 2, Waltham, MA, USA) operating at 20 kV after gold sputtering. The hydrophilicity of the PEN and epoxy coating surfaces was characterized via a contact angle measuring system (KRUSS Scientific Instruments (Shanghai) Co., Ltd., DSA 30, Shanghai, China) at ambient temperature. Contact angle values were obtained by analyzing the droplet shape with the system’s software. For each sample, measurements were taken at three distinct points, and the reported results are the average of these measurements. The barrier performances of the PEN and epoxy coatings with various molecular weights were assessed with a gas permeability tester (Jinan SYSTESTER Testing Technology Co., Ltd., GTR-701R, Jinan, China) at ambient pressure, using air as the permeant. The film thickness was 65 ± 5 µm.

#### 2.4.3. Electrochemical Testing

Protective performance of the PEN and epoxy coatings was evaluated in terms of both EIS and potentiodynamic polarization measurements via an electrochemical workstation (Wuhan CorrTest Instruments Co., Ltd., CS2350M, Wuhan, China). All tests were conducted in a 3.5 wt% NaCl solution with a three-electrode cell, featuring the coated sample (exposed area of 1 cm^2^) as the working electrode, a platinum sheet (LeiCi Instrument Factory, Leici213-01, Shanghai, China) as the counter electrode and a mercurous chloride electrode (LeiCi Instrument Factory, Leici232-01, Shanghai, China) as the reference electrode. EIS data were acquired over a frequency range of 10^5^ Hz to 10^−2^ Hz with a 10 mV AC perturbation. Potentiodynamic polarization scans were performed from −250 mV to +250 mV relative to the open circuit potential at a rate of 1 mV/s.

#### 2.4.4. Salt Spray Test of Ep and PEN Coatings

Prolonged anti-corrosion performance of the PEN and epoxy coatings was assessed via neutral salt spray tests following the ASTM B117 standard [31]. An “X”-shaped scratch (50 mm × 50 mm) was introduced onto each coating surface with a cutter. Subsequently, the scribed samples, positioned at a 45° angle, were subjected to a 5 wt% NaCl salt spray atmosphere at 25 ± 1 °C inside a test chamber (Red Samarium Instrument Technology (Shenzhen) Co., Ltd., 60D, Shenzhen, China). The progression of corrosion upon exposure was monitored and photographed at various time intervals.

## 3. Results

### 3.1. Characterization of PEN Material with Different Molecular Weights

**Figure 2:**
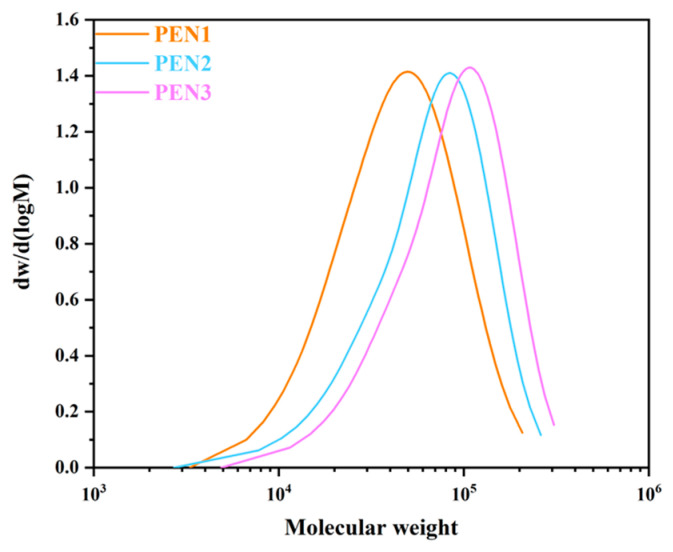
GPC curves of PEN materials synthesized with different polymerization times.

The gel permeation chromatograph (GPC) analysis confirmed the molecular weight evolution of PEN with increasing polymerization time, with the results plotted in Figure 2 and the parameters obtained from curves summarized in Table 3. As depicted in Figure 2, all samples exhibited a unimodal molecular weight distribution, which signified polymer formation with relatively homogeneous chain lengths. PEN1 displayed the lowest molecular weight range from 3328 to 207,144, with an M_w_ of 55,870 and an M_n_ of 30,743. With the polymerization time extended to 1.5 h, PEN2 broadened the range to 3916–261,103, acquiring an M_w_ of 82,336 and an M_n_ of 46,838. The highest molecular weight was seen for PEN3 after 2 h, with a range of 4890–306,729 and M_w_/M_n_ values of 103,089/61,719. Additionally, the polydispersity indices (PDI) for PEN1, PEN2, and PEN3 were 1.749, 1.758, and 1.670, respectively. The narrow and consistent PDI values indicate that the polymerization activity remained nearly unchanged despite the prolonged reaction time.

**Figure 3:**
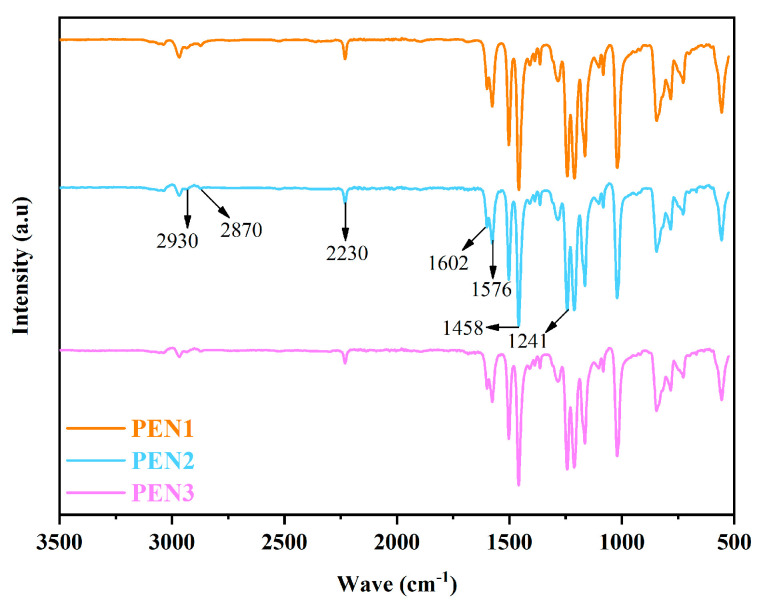
FTIR spectra obtained for PEN with different polymerization times.

Preliminary confirmation of the chemical structures of the PEN samples with differing molecular weights was achieved through FTIR analysis. As illustrated in Figure 3, the relatively weak absorption bands located at 2930 and 2870 cm^−1^ were associated with the C-H stretching vibrations originating from the methyl groups within the BPA structural moieties. The absorption feature at 2230 cm^−1^ arose from the -C≡N stretching vibration of the cyano substituents on the aromatic rings in the PEN framework. Additionally, the absorption signals observed at 1457, 1576, and 1601 cm^−1^ were attributable to the characteristic vibrations of the benzene ring units. The band at 1242 cm^−1^ corresponded to the aromatic ether linkages generated via the condensation reaction between BPA and DCBN. The absence of peak shifts or disappearance across all samples indicated that the three PEN samples with different molecular weights shared the same chemical structure.

**Figure 4:**
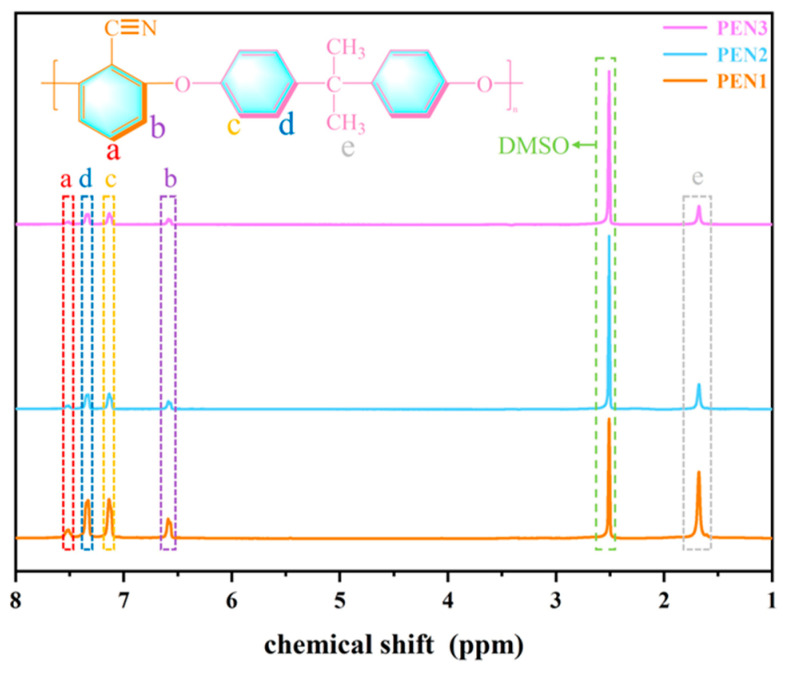
^1^H-NMR spectra of PEN with different molecular weights.

The successful synthesis of PEN was confirmed by qualitative ^1^H-NMR analysis. As observed in Figure 4, all PEN samples exhibited consistent peak patterns without any shifts or missing signals. Specifically, the resonances at 7.07–7.21 ppm and 7.27–7.43 ppm can be ascribed to the aromatic protons (c and d) originating from the bisphenol A (BPA) moiety, while the signal at 1.56–1.79 ppm (signal e) corresponds to its methyl protons. The characteristic signals at 6.53–6.65 ppm (signal b) and 7.47–7.59 ppm (signal a) are characteristic of the two distinct aromatic protons in the DCBN unit. The prominent peak at 2.47–2.57 ppm is attributed to the DMSO-d_6_ solvent [32,33]. The ^1^H-NMR data, in conjunction with the FTIR results, provides unambiguous evidence for the successful preparation of PEN with varying molecular weights.

**Figure 5:**
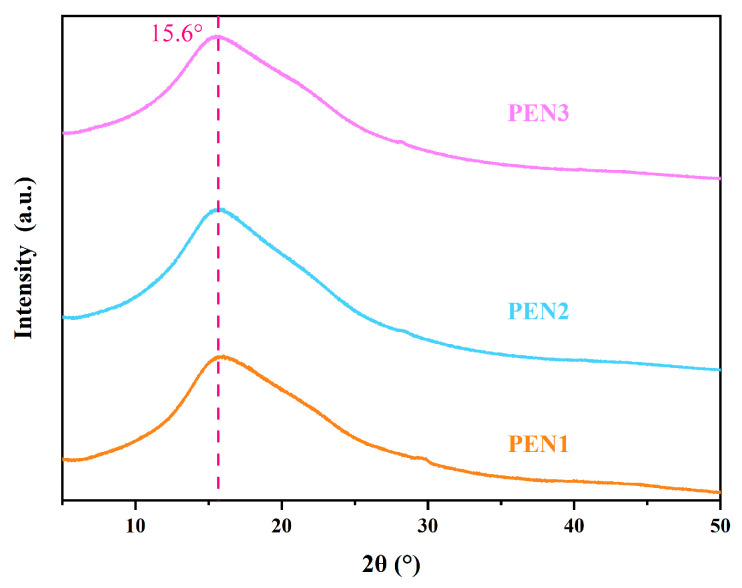
XRD patterns of PEN samples with varying molecular weights.

The amorphous nature of PEN with varying molecular weights was confirmed by XRD analysis in Figure 5. All of the PEN samples displayed a characteristic broad amorphous hump centered at around 15.6° (2θ), and no crystalline diffraction peaks were detected, demonstrating the amorphous nature of the synthesized PEN irrespective of molecular weight.

**Figure 6:**
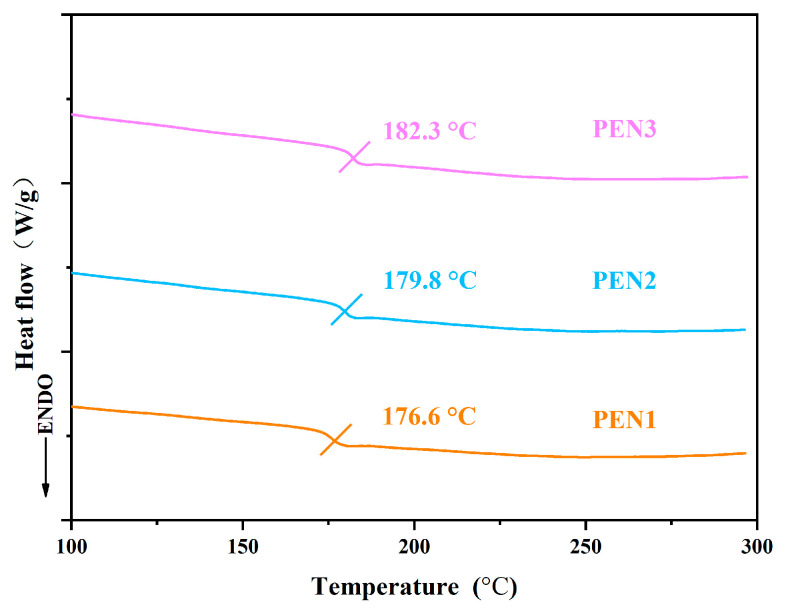
DSC thermograms obtained for PEN with different molecular weights.

DSC analysis was performed to evaluate the glass transition temperatures (T_g_) of PEN with various molecular weights. The results, shown in Figure 6, indicate that all PEN samples possessed T_g_ values exceeding 175 °C, confirming their adequate thermal resistance. A clear dependence of T_g_ on molecular weight was observed: the T_g_ increased from 176.6 °C for PEN1 to 182.3 °C for PEN3. This enhancement was attributed to the longer chain segments in higher-molecular-weight PEN, which promoted stronger interchain interactions. As a result, greater thermal energy was required to overcome these interactions and activate segmental motion, leading to an elevated T_g_ as detected by DSC.

### 3.2. Characterization of Epoxy and PEN Coatings

#### 3.2.1. Thermal Resistances of Ep and PEN Coatings

**Figure 7:**
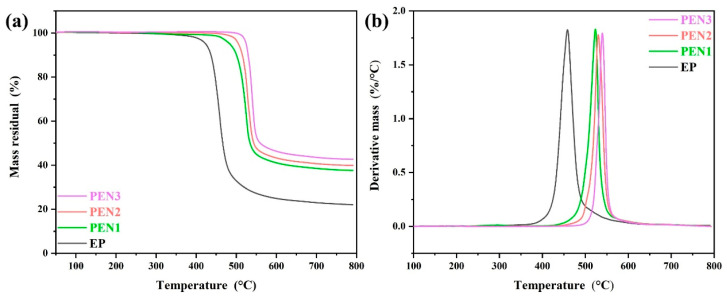
The TGA and DTG curves of EP and different PEN coatings. (**a**) TGA; (**b**) DTG.

Thermal stabilities of the epoxy and PEN coatings were evaluated by thermogravimetric analysis (TGA), with the results shown in Figure 7. As shown in Figure 7a, the epoxy coating exhibited inferior thermal stability compared to PEN, showing a 5% weight loss temperature (T_5%_) at 360 °C and a maximum decomposition rate temperature (T_max_) at 443 °C. In contrast, the PEN coatings exhibited T_5%_ and T_max_ values above 450 °C and 500 °C, respectively. Furthermore, the derivative thermogravimetry (DTG) curves revealed that with increasing molecular weight (Figure 7b), the T_5%_ of PEN increased from 480 °C to 521 °C, and the T_max_ shifted from 523 °C to 540 °C. This enhancement can be contributed to the PEN with higher molecular weight possessing increased molecular chain length that strengthened the interchain interactions. Consequently, a higher temperature was required to disrupt the PEN’s chain structure, leading to improved heat resistance. The above results demonstrate that PEN possesses superior thermal stability compared to EP, and PEN could effectively enhance its thermal resistance with increments in molecular weight.

#### 3.2.2. The Mechanical Properties of Ep and PEN Coatings

**Figure 8:**
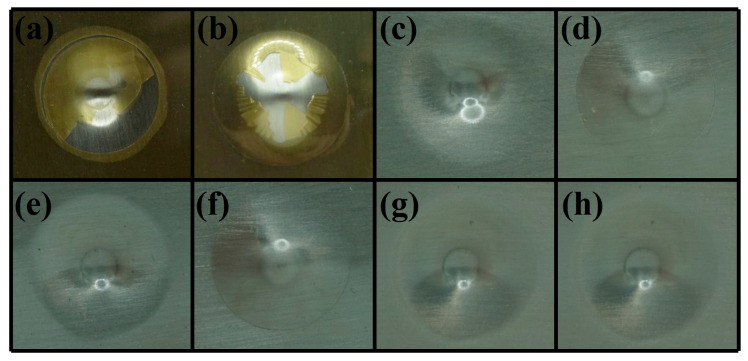
Impact test diagrams of EP and different coatings. (**a**) EP’s direct impact; (**b**) EP’s reverse impact; (**c**) PEN1’s direct impact; (**d**) PEN1’s reverse impact; (**e**) PEN2’s direct impact; (**f**) PEN2’s reverse impact; (**g**) PEN3’s direct impact; (**h**) PEN3’s reverse impact.

Impact strength serves as vital assessment criteria for coating toughness. Therefore, the resistance of EP and different PEN coatings to rapid deformation under external force was evaluated via impact experiments. As depicted in Figure 8, both the direct and reverse impact specimens of epoxy coating showed a critical delamination phenomenon, demonstrating its poor resistance to rapid deformation and its inferior toughness. This can be attributed to the intricate 3D crosslinked network structure formed upon bisphenol A epoxy resin’s curing process. The rigid benzene rings on chain segments were mutually constrained, hindering the momentary stress from dissipating through the restricted segments upon impact, leading to stress concentration and subsequent brittle fracture of EP. In comparison, for PEN1, PEN2 and PEN3, all the direct and reverse impact specimens remained intact on metal substrates without any delamination observed, demonstrating PEN’s excellent impact resistance. The remarkable performance stems from the fact that although PEN contained rigid benzene rings on structure units, its linear nature and the presence of flexible ether oxygen linkages in PEN’s main chain allowed the force to dissipate through the segment’s structure, rapidly dispersing the energy and thus exhibiting outstanding toughness.

**Figure 9:**
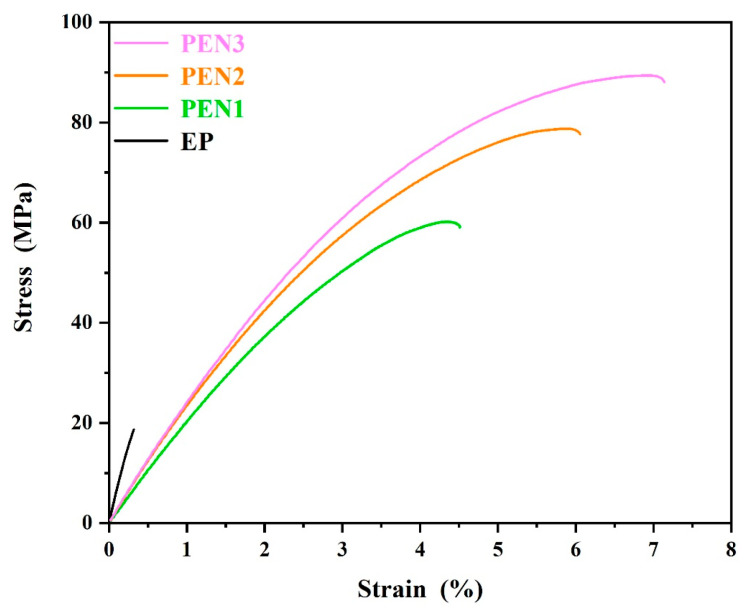
Tensile strength curves of EP and different PEN coatings.

The tensile strength capacity of the epoxy and PEN coatings was confirmed via tensile testing, with the corresponding stress–strain curves plotted in Figure 9. The results reveal a transition from brittle to ductile fracture behavior from the EP coating to the PEN coatings. The EP coating displayed typical characteristics of brittle materials when it fractured abruptly after limited deformation, exhibiting a tensile strength and elongation at break of 18.67 MPa and 0.32%, respectively. Conversely, all of the PEN coatings exhibited typical ductile fracture, featuring distinct yield behavior and deformation quantities. According to quantitative analysis, PEN1, PEN2 and PEN3 presented tensile strengths of 60.17 MPa, 78.74 MPa and 89.37 MPa, respectively, with corresponding elongations at break of 4.51%, 6.06%, and 7.14%, respectively. The PEN coatings’ superior tensile properties originate from their molecular structures, combining rigid aromatic rings to supply mechanical robustness with flexible ether linkages for energy dissipation. This enabled PEN to effectively relieve stress through segmental motion, avoiding the stress concentration that led to brittle failure in the epoxy crosslinked network. In addition, the PEN’s tensile properties were confirmed to be strongly dependent on molecular weight, which was evidenced by the simultaneous improvements in tensile strength and elongation with increasing molecular weight. This improvement was attributed to increased interchain entanglement and stronger intermolecular forces in longer polymer chains that imparted greater energy absorption capacity and required greater stresses to disrupt chain interactions.

**Figure 10:**
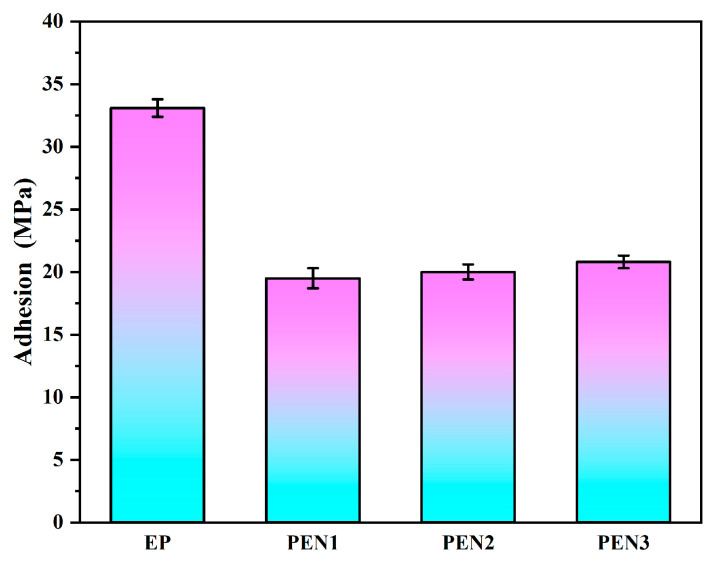
Pull-off adhesion results of EP and different PEN coatings.

The adhesion properties of the EP coating and the PEN coatings with varying molecular weights on metal substrates were assessed via pull-off testing. As seen from Figure 10, the results reveal that the adhesion strengths of PEN1, PEN2, and PEN3 were 19.5 MPa, 20.0 MPa, and 20.8 MPa, respectively, presenting comparable values regardless of molecular weight. The EP coating exhibited a substantially higher adhesion strength of 34.2 MPa. This disparity stemmed from the PEN’s coordination interactions between the lateral cyano groups and metal surface that supplemented its weak chemical bond force relative to EP, despite the EP and PEN coatings’ common physical anchoring forces in adhering to the metal substrate. And the superior adhesion of EP arose from the formation of strong covalent chemical bonds via epoxy groups’ ring-opening reactions with the metal substrate during curing. This chemical bonding significantly enhanced the interfacial adhesion of EP to the metal surface.

**Figure 11:**
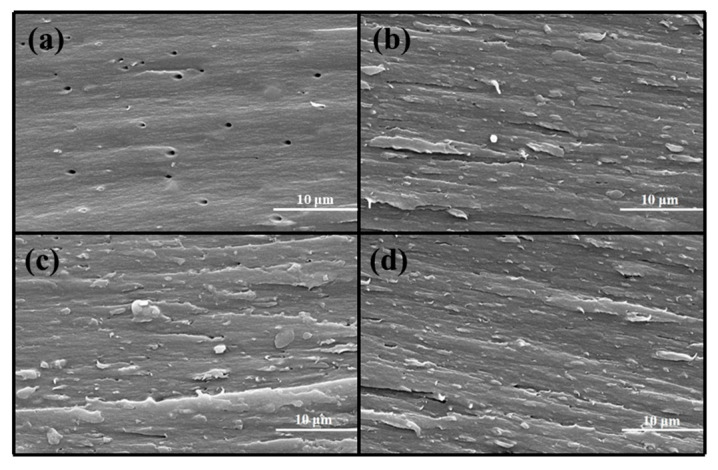
SEM images of EP and PEN coating’s fracture surfaces after tensile testing: (**a**) EP; (**b**) PEN1; (**c**) PEN2; and (**d**) PEN3.

The fracture surfaces of the EP and PEN coatings after tensile failure were examined by SEM (Figure 11). As observed in Figure 11a, the EP sample revealed a smooth, featureless fracture surface with several micro-voids observed on it, which was consistent with typical brittle fracture features. In stark contrast, the PEN coatings (Figure 11b–d) exhibited rough and tortuous fracture surfaces with pronounced crack deflection and evident plastic deformation. Moreover, fewer micro-voids with diminished dimensions were observed in the PEN coatings than in the epoxy coating. This denser morphology offered additional evidence supporting the gas permeability measurements.

#### 3.2.3. Water Contact Angle Measurements of EP and PEN Coatings

**Figure 12:**
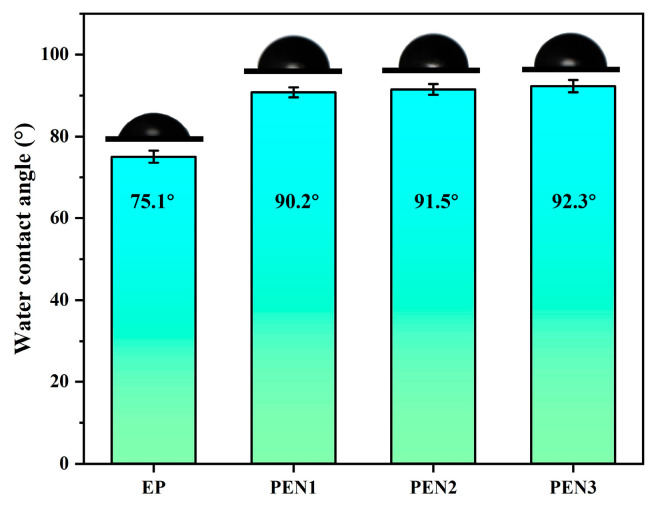
Water contact angle profiles of EP and PEN coatings.

Hydrophobicity serves as an important parameter to evaluating a coating material’s resistance to water, as coatings with good hydrophobic capacities can effectively mitigate the corrosion of metal substrates by corrosive media. Therefore, the hydrophobicity of the EP and PEN coatings was assessed by water contact angle measurements, and the concerning digital images are displayed in Figure 12. As shown in the images, the water contact angles of PEN1, PEN2, and PEN3 were 90.8°, 91.5° and 92.3°, respectively, exhibiting minimal variation with molecular weight. For the EP coating, it displayed a water contact angle of 75.1°, which indicated inferior hydrophobicity compared to the PEN coatings. These results can be attributed to the presence of hydrophilic groups (such as hydroxyl and epoxy groups) within the EP coating structure that enhanced the affinity of the EP coating for water, thereby resulting in its lower hydrophobicity relative to the PEN coatings.

#### 3.2.4. Air Permeability Measurements of EP and PEN Coatings

**Figure 13:**
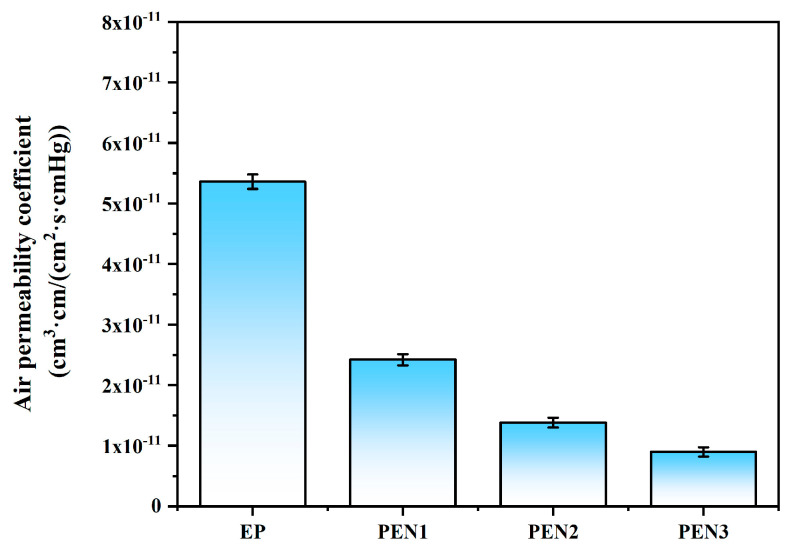
Air permeability coefficients diagrams of EP and PEN coatings.

The compactness of the EP and PEN coatings was checked through air permeability measurements (Figure 13). The results demonstrate that the EP coating possessed an air permeability coefficient of 5.36 × 10^−11^ cm^3^·cm/(cm^2^·s·cmHg), which was considerably higher than those of the PEN coatings (2.42 × 10^−11^, 1.28 × 10^−11^ and 8.97 × 10^−12^ cm^3^·cm/(cm^2^·s·cmHg) for PEN1, PEN2 and PEN3). These comparative results convincingly prove the PEN coatings’ superior compactness. Additionally, the air permeability coefficient of the PEN coatings showed a clear molecular weight dependence, decreasing progressively with increments in molecular weight. This trend indicates that higher molecular weights contributed to the formation of more compact PEN coatings with enhanced barrier properties.

#### 3.2.5. Water Absorption Measurements of EP and PEN Coatings

**Figure 14:**
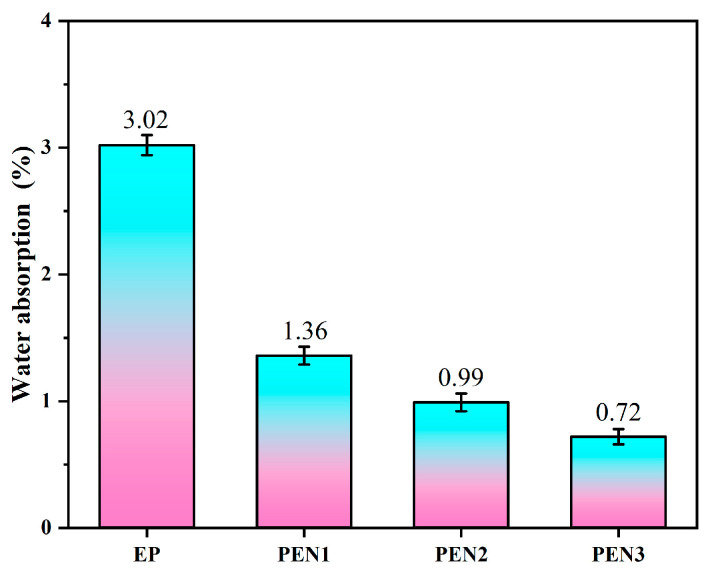
Water absorption rates diagram of EP and PEN coatings.

The resistances of the EP and PEN coatings against water were further evaluated by immersing the samples in water for 48 h, followed by air-drying and the weighing process. As shown in Figure 14, the water absorption rates of the EP, PEN1, PEN2 and PEN3 coatings after 48 h of immersion were 3.02%, 1.36%, 0.99%, and 0.72%, respectively. Compared to the PEN coatings, EP exhibited significantly higher water absorption. Integrating the findings with the results obtained from the contact angle and air permeability measurements, the inferior water resistance of the EP coating could be ascribed to two synergistic factors: the lower hydrophobicity arose from the presence of hydrophilic groups within its structure and its relatively poorer compactness compared to the PEN coatings.

### 3.3. Electrochemical Measurements

#### 3.3.1. EIS Measurements of EP and PEN Coatings

Corrosion protection performance of the EP and PEN coatings was primarily investigated by electrochemical impedance spectroscopy (EIS). Prior to the test, the EP, PEN1, PEN2 and PEN3 coated samples were immersed in a 3.5 wt% NaCl electrolyte until a stable open circuit potential was achieved. EIS spectra, which included Nyquist and Bode plots, were then recorded at various time intervals to monitor each coating’s performance evolution. Generally, the radius of the capacitive arc in the Nyquist plots denoted a qualitative measure of coating protection; a larger radius signified superior barrier properties against corrosive media under equivalent conditions [34]. Additionally, the impedance modulus at the lowest frequency of 0.01 Hz (|Z|_0.01Hz_) in the Bode diagram was deemed as a semi-quantitative indicator of barrier performance [35]. Furthermore, the shift in phase angle and reduction in the high-angle phase range reflected the coating’s deviation from ideal capacitive behavior, which was caused by electrolyte diffusion toward the substrate, with greater deviation indicating more severe corrosion [36].

**Figure 15:**
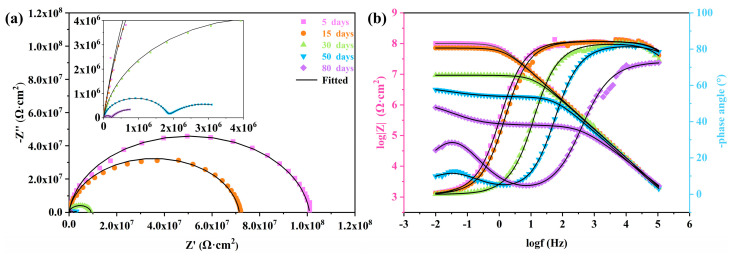

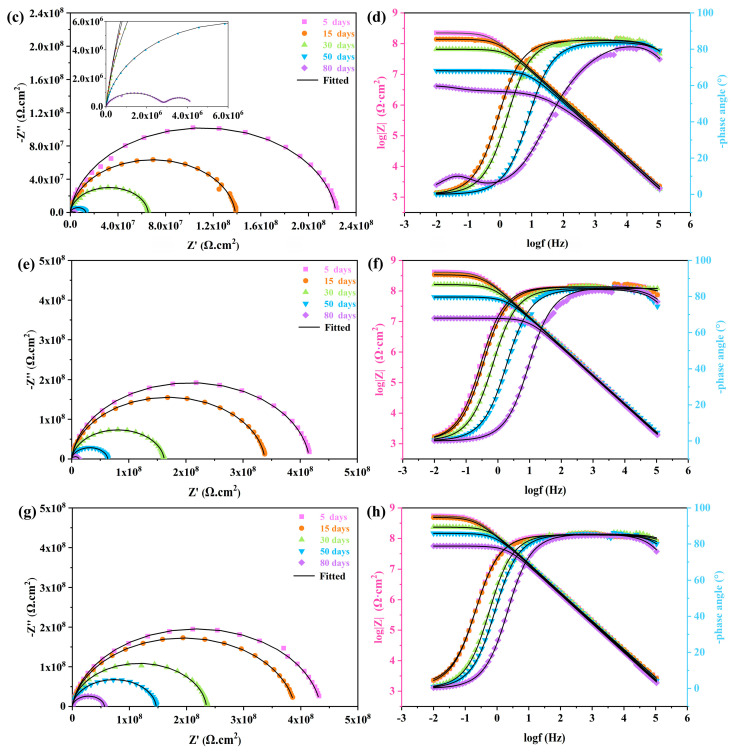
EIS spectra of EP and different PEN coatings: (**a**) Nyquist plots of EP; (**b**) Bode plots of EP; (**c**) Nyquist plots of PEN1; (**d**) Bode plots of PEN1; (**e**) Nyquist plots of PEN2; (**f**) Bode plots of PEN2; (**g**) Nyquist plots of PEN3; (**h**) Bode plots of PEN3.

As depicted in Figure 15a,b, the EP coating exhibits a progressive decrease in its capacitive arc radius over time. The |Z|_0.01Hz_ value decreased from 1.012 × 10^8^ Ω·cm^2^ after 5 days to 9.323 × 10^6^ Ω·cm^2^ after 30 days, while the onset frequency of the high phase angle region showed a shift from 1 Hz to 745 Hz. This degradation is ascribed to the gradual decline in barrier performance with electrolyte permeation [37]. As the time extended to 50 days, the EP displayed two distinct capacitive arcs in the Nyquist plots, and a small peak emerged in the phase angle plots within 1 Hz to 10^−2^ Hz, which corresponds to the corrosion response of the metal substrate [38]. This indicates that the corrosive medium had penetrated to the coating/substrate interface, leading to delamination and failure of the EP coating. In contrast, as presented in Figure 15c,d, the PEN1 coating retained a single capacitive arc in its Nyquist plot after 50 days of immersion, accompanied by a single broad peak in the phase angle plot. The |Z|_0.01Hz_ value for PEN1 at this time was 1.314 × 10^7^ Ω·cm^2^, considerably higher than that of the failed EP coating (3.090 × 10^6^ Ω·cm^2^), demonstrating the superior corrosion resistance of PEN1. However, after 80 days, PEN1 eventually failed due to electrolyte penetration reaching the substrate, as evidenced by the appearance of two capacitive arcs in its Nyquist plots and a low-frequency corrosion response peak in its phase angle plot.

As illustrated in Figure 15e–h, both the PEN2 and PEN3 coatings maintained their integrity throughout the entire immersion period without failure. Their Nyquist plots consistently exhibit a single capacitive arc, and their phase angle plots show no corrosion response peaks, retaining the characteristic features of a single time constant system. Notably, the |Z|_0.01Hz_ value for the PEN3 coating after 80 days was 5.665 × 10^7^ Ω·cm^2^, significantly higher than that of PEN2 (1.287 × 10^7^ Ω·cm^2^). These results demonstrate that the PEN3 coating possessed the most superior and durable corrosion resistance among all samples tested, with higher molecular weight correlating with enhanced barrier performance.

**Figure 16:**
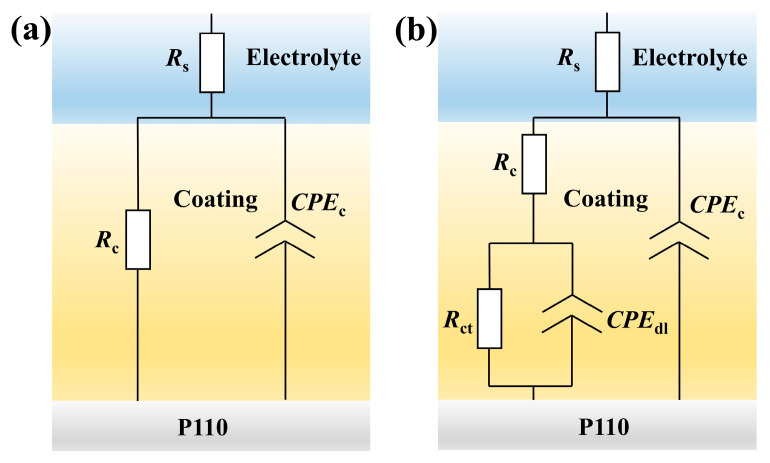
Equivalent circuit models for fitting EIS data of EP and PEN coatings: (**a**) R(QR) model; (**b**) R(RQ(RQ)) model.

To further elucidate the EIS results obtained from the EP and different PEN coatings, the impedance spectra were fitted with ZSimpWin (Version3.30) software’s appropriate equivalent circuit model (Figure 16). The corresponding electrochemical parameters derived from the fitting are summarized in Table 4. Figure 16a presents the R(QR) equivalent circuit model, which is applicable to coating systems exhibiting a single time constant during the initial stage of corrosion [39]. This circuit comprised the following parameters: solution resistance (R_s_), coating resistance (R_c_), and constant phase element of the coating (CPE_c_). Both R_c_ and CPE_c_ served as indicators of the coating’s barrier properties. Generally, a higher R_c_ value signifies superior barrier performance against corrosive media, whereas a larger CPEc value indicates a greater deviation of the coating from ideal capacitive behavior. Figure 16b illustrates the R(RQ(RQ)) equivalent circuit model, which was suitable for systems that developed a two-time constant due to coating failure in the intermediate stage of corrosion [40]. Compared to the R(QR) model, the circuit introduces two additional parameters: charge transfer resistance (R_ct_) and constant phase element of the double layer (CPE_dl_). R_ct_ represents the resistance to electron transfer across the interface between coating and electrolyte, with higher values indicating greater difficulty in electron migration. CPE_dl_ reflects the charge distribution at the interface between coating and electrolyte, with larger values suggesting increased charge accumulation at the interface.

As summarized in Table 4, all coatings exhibit a progressive decrement in R_c_ value and a corresponding increment in CPE_c_ value with the immersion time prolonged. This trend indicates the gradual diffusion of corrosive media into the coatings, leading to deteriorated barrier capacities and an increasing deviation from ideal capacitive behavior. Among them, the EP coating demonstrated inferior barrier performance compared to the PEN coatings, with its R_c_ value declining to 9.260 × 10^6^ Ω·cm^2^ and its CPEc increasing to 6.323 × 10^−9^ Ω^−1^·cm^−2^·s^n^ after 30 days. As the time reached 50 days, the emergence of the R_ct_ and CPE_dl_ parameters for EP confirmed the EP coating’s failure. For the PEN1 coating, it maintained a single time constant until 80 days, at which point R_ct_ and CPE_dl_ parameters appeared, indicating eventual failure. Furthermore, PEN1 exhibited a higher R_c_ (2.827 × 10^6^ Ω·cm^2^) and R_ct_ (4.148 × 10^6^ Ω·cm^2^) through 80 days of the test, along with lower CPE_c_ (1.346 × 10^−8^ Ω^−1^·cm^−2^·s^n^) and CPE_dl_ (1.265 × 10^−6^ Ω^−1^·cm^−2^·s^n^) values compared to the failed EP coating at 50 days, demonstrating its superior corrosion resistance. The trend of higher R_c_ and lower CPE_c_ for PEN1 relative to EP was consistently observed at all preceding time points.

For the PEN2 and PEN3 coatings, no parameters corresponding to a two-time constant system appeared throughout the entire immersion period, indicating their superior barrier performance compared to PEN1. Further comparison revealed that after 80 days, PEN3 possessed a higher R_c_ value (5.647 × 10^7^ Ω·cm^2^) and a lower CPE_c_ value (3.029 × 10^−9^ Ω^−1^·cm^−2^·s^n^) than PEN2, demonstrating that PEN3 exhibited the best corrosion resistance among all coatings tested.

#### 3.3.2. Polarization Measurements of EP and PEN Coatings

**Figure 17:**
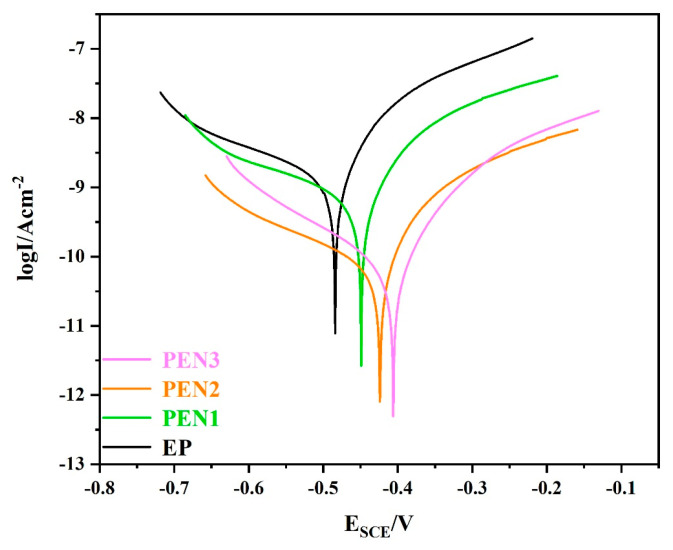
Comparative potentiodynamic polarization behaviors of EP and PEN coatings.

Corrosion resistance of the EP and PEN coatings after 30 days of immersion in 3.5 wt% NaCl solution was further assessed by potentiodynamic polarization measurements (Figure 17). The corresponding electrochemical parameters derived from Tafel extrapolation are summarized in Table 5. The EP coating exhibited the most negative corrosion potential (E_corr_ = −0.484 V) and the highest corrosion current density (I_corr_ = 1.180 × 10^−9^ A/cm^2^), indicating its inferior barrier properties. In contrast, all of the PEN coatings showed more positive E_corr_ values and lower I_corr_ values. Among them, PEN3 demonstrated the best corrosion resistance, with the most positive E_corr_ (−0.403 V) and the lowest I_corr_ (6.345 × 10^−11^ A/cm^2^). These findings are consistent with the EIS results, confirming that increasing molecular weight enhanced the corrosion protection performance of the PEN coatings.

#### 3.3.3. Salt Spray Test of EP and PEN Coatings

**Figure 18:**
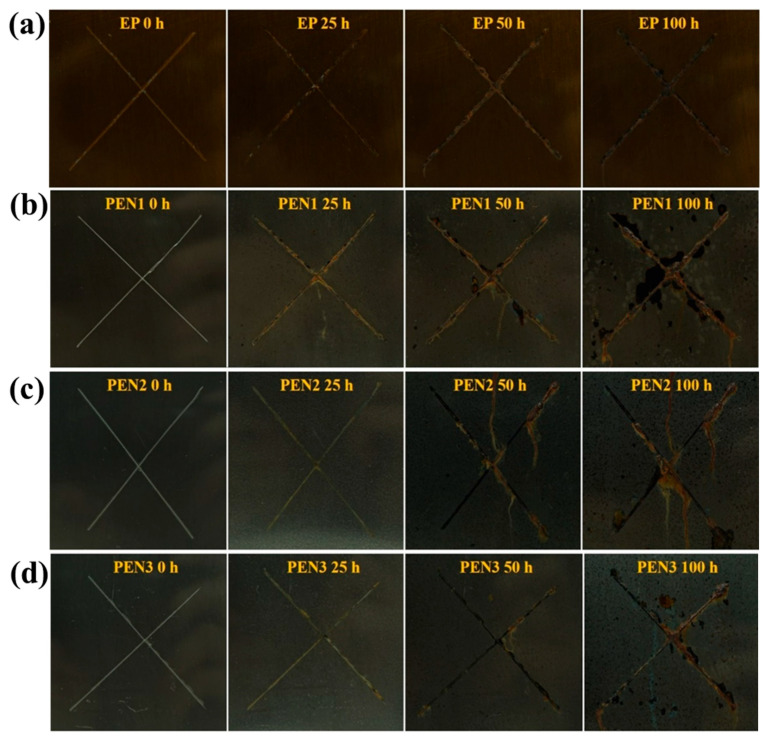
Macroscopic morphologies of EP and PEN coatings after salt spray testing: (**a**) EP, (**b**) PEN1, (**c**) PEN2, and (**d**) PEN3.

Long-term corrosion protection performance of the EP and PEN coatings was assessed by neutral salt spray testing over 100 h following the ASTM B117 standard in Figure 18. After 100 h of exposure, the EP coating (Figure 18a) exhibited good corrosion resistance, with only localized corrosion products near the scratch and no evidence of diffusion. In contrast, all of the PEN coatings showed corrosion product diffusion from the scratch after 50 h (Figure 18b–d). This was attributed to the weaker physical adhesion of PEN to the metal substrate compared to EP, which allowed the corrosive medium to more rapidly compromise the interface and initiate corrosion reactions. At 50 h, PEN1 and PEN2 exhibited comparable diffusion, while PEN3 showed slightly less propagation. After 100 h, PEN1 displayed extensive black corrosion product diffusion from the scratch accompanied by blistering, indicating poor salt spray resistance. The PEN2 and PEN3 coatings maintained their integrity without blistering and exhibited less corrosion diffusion than PEN1. PEN3 showed the least propagation among the three PEN coatings. In summary, EP exhibited the best salt spray resistance, followed by PEN3, PEN2, and PEN1 in descending order (PEN3 > PEN2 > PEN1). Notably, this ranking differs from the trend observed in the EIS and polarization measurements, where PEN coatings outperformed EP, suggesting that different corrosion mechanisms may dominate under salt spray conditions.

#### 3.3.4. Investigation of Coating Failure Mechanisms

**Figure 19:**
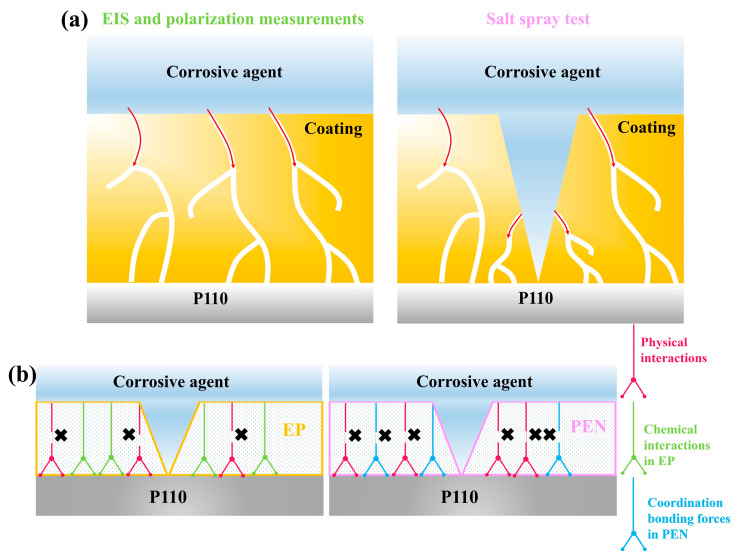
Schematic illustrations of coating failure mechanisms: (**a**) comparison of corrosion pathways in EIS/polarization testing (intact coating) versus salt spray testing (scribed coating); (**b**) comparison of interfacial bonding mechanisms between EP and PEN coatings on metal substrates.

The differing trends observed between salt spray testing and electrochemical measurements (EIS and polarization) were investigated by examining the respective failure mechanisms (Figure 19). For EIS and polarization testing, the coatings were intact and defect free (Figure 19a). Corrosive media must permeate through micropores to reach the substrate, a process effectively hindered by the higher compactness and hydrophobicity of the PEN coatings, leading to their superior performance in these tests. In salt spray testing, however, the coatings were deliberately scribed, creating artificial defects. These defects allowed direct ingress of corrosive media to the coating/substrate interface, accelerating failure. The physical adhesion forces from mechanical anchoring are weak and easily disrupted by the electrolyte. By comparison, chemical bonding at the interface was stronger and more resistant to degradation. As illustrated in Figure 19b, EP formed strong covalent bonds on the metal substrate via its epoxy and hydroxyl groups, whereas PEN only established weak coordination interactions through its lateral cyano groups. Therefore, when physical adhesion had deteriorated, the robust chemical bonds in EP maintained interfacial integrity and prevented delamination with the metal surface. On the other hand, the weaker coordination bonds in PEN were insufficient to prevent detachment, resulting in accelerated substrate corrosion. This mechanism difference accounted for the superior salt spray resistance of EP compared to PEN, despite the reverse ranking observed in electrochemical tests on intact coatings.

## 4. Conclusions

In this study, a series of bisphenol A-based polyarylene ether nitrile (PEN) materials with different molecular weights and excellent solubility were synthesized by adjusting the polymerization time. Following fundamental characterization, coating samples were fabricated using the synthesized PEN and epoxy resin (EP) for comparative evaluation of their water resistance, thermal stability, mechanical capacities and corrosion resistance. The main findings are summarized as follows:

(1) Contributing to the intrinsic compactness and better hydrophobic property, the water resistances of the PEN coatings were comparatively better than EP, which indicated that PEN offers better barrier capacity against moisture penetration. Moreover, a positive correlation was observed between the molecular weight of PEN and its water resistance, with higher molecular weights leading to enhanced performance. This confirmed that PEN coatings with higher molecular weights possess denser molecular structures.
(2) Thermogravimetric analysis showed that the PEN coatings possessed higher maximum decomposition rate temperature in contrast to the EP coating, denoting outstanding thermal stability of PEN. Among them, the high-molecular-weight PEN3 exhibited a 5% weight loss temperature (T_5%_) of 521 °C and a maximum decomposition temperature (T_max_) of 540 °C, representing increases of 44.72% and 21.90%, respectively, compared to EP. This enhancement could be explained by the higher molecular weight of PEN providing extended chain lengths that intensified interchain bonding. Consequently, the thermal energy needed to destabilize the chain architecture increased.
(3) In terms of mechanical behavior, the PEN coatings showed significantly improved tensile strength, elongation at break, and impact toughness relative to EP. However, due to the absence of strong chemical bonds at the coating/substrate interface in contrast to EP, their adhesion strengths were approximately 20 MPa, about 14 MPa lower than that observed for EP. Additionally, the salt spray test findings correlate well with the earlier discussion, further validating the proposed coating failure mechanisms in this study. Thus, further enhancing PEN adhesion to metal substrates is needed in future research.
(4) Electrochemical evaluations revealed that all the PEN coatings offered better corrosion protection than EP. This was ascribed to better intrinsic compactness and hydrophobic properties of PEN that led to a more tortuous diffusion path for corrosive species within the coating. In particular, PEN3, with the densest molecular structure, displayed the best performance.

## Figures and Tables

**Table 1:** The chemical reagents used in this study.

Reagent	Formula	Purity	Manufacturer
2,6-Dichlorobenzamide	C_7_H_3_Cl_2_N	≥98%	Aladin, Shanghai, China
Bisphenol A	C_15_H_16_O_2_	≥99%	Aladin, Shanghai, China
potassium carbonate	K_2_CO_3_	AR, ≥99%	Aladin, Shanghai, China
E-51 epoxy resin	/	TP	China Bluestar Chengrand Co., Ltd., Chengdu, China
sodium chloride	NaCl	AR, ≥99.5%	Kelong Chemical Reagent Factory, Chengdu, China
Toluene	C_7_H_8_	AR, ≥99.5%	Kelong Chemical Reagent Factory, Chengdu, China
Cyclohexanone	C_6_H_10_O	AR, ≥99.5%	Kelong Chemical Reagent Factory, Chengdu, China
Ethanol	C_2_H_5_OH	AR, ≥95%	Kelong Chemical Reagent Factory, Chengdu, China
N-methylpyrrolidone	C_5_H_9_NO	AR, ≥99%	Kelong Chemical Reagent Factory, Chengdu, China
Hydrochloric acid	HCl	AR, 36–38%	Kelong Chemical Reagent Factory, Chengdu, China
Deionized water	H_2_O	UP	Laboratory produced

**Table 2:** Synthesis conditions and molecular weight parameters of PEN samples.

Sample	Dehydration Time	Temperature	Time
PEN1	2 h	200 °C	1 h
PEN2	2 h	200 °C	1.5 h
PEN3	2 h	200 °C	2 h

**Table 3:** Detailed parameters of PEN samples of different molecular weights.

Sample	M_w_	M_n_	PDI
PEN1	55,870	30,743	1.749
PEN2	82,336	46,838	1.758
PEN3	103,089	61,719	1.670

**Table 4:** Fitted electrochemical parameters of EP and PEN coatings from EIS analysis.

	Time (Day)	*CPE*_c_		*R*_c_ (Ω·cm^2^)	*CPE*_dl_		*R*_ct_ (Ω·cm^2^)
		Y_0_ (Ω^−1^·cm^−2^·s^n^)	n_coat_		Y_0_ (Ω^−1^·cm^−2^·s^n^)	n_dl_	
EP	5	1.647 × 10^−9^	0.94	1.009 × 10^8^	—	—	—
	15	2.895 × 10^−9^	0.93	7.145 × 10^8^	—	—	—
	30	6.323 × 10^−9^	0.92	9.260 × 10^6^	—	—	—
	50	3.541 × 10^−8^	0.85	1.836 × 10^6^	1.962 × 10^−6^	0.63	3.125 × 10^6^
	80	9.040 × 10^−8^	0.78	2.214 × 10^5^	5.348 × 10^−6^	0.59	7.533 × 10^5^
PEN1	5	9.239 × 10^−10^	0.94	2.230 × 10^8^	—	—	—
	15	1.445 × 10^−9^	0.94	1.386 × 10^8^	—	—	—
	30	4.632 × 10^−9^	0.93	6.543 × 10^7^	—	—	—
	50	8.123 × 10^−9^	0.90	1.309 × 10^7^	—	—	—
	80	1.346 × 10^−8^	0.85	2.827 × 10^6^	1.265 × 10^−6^	0.66	4.148 × 10^6^
PEN2	5	5.964 × 10^−10^	0.95	4.161 × 10^8^	—	—	—
	15	7.048 × 10^−10^	0.95	3.382 × 10^8^	—	—	—
	30	9.797 × 10^−10^	0.94	1.616 × 10^8^	—	—	—
	50	4.263 × 10^−9^	0.93	6.353 × 10^7^	—	—	—
	80	7.966 × 10^−9^	0.91	1.280 × 10^7^	—	—	—
PEN3	5	4.462 × 10^−10^	0.96	4.348 × 10^8^	—	—	—
	15	5.578 × 10^−10^	0.95	3.895 × 10^8^	—	—	—
	30	7.963 × 10^−10^	0.95	2.348 × 10^8^	—	—	—
	50	9.875 × 10^−10^	0.94	1.468 × 10^8^	—	—	—
	80	3.029 × 10^−9^	0.93	5.647 × 10^7^	—	—	—

**Table 5:** Electrochemical parameters derived from EIS fittings for EP and PEN coatings.

Sample	*E*_corr_ (V vs. SCE)	*I*_corr_ (A/cm^2^)	*β*_a_ (mv)	*β*_c_ (mv)
EP	−0.484	1.180 × 10^−9^	84.63	120.17
PEN1	−0.450	4.792 × 10^−10^	77.06	102.65
PEN2	−0.423	7.349 × 10^−11^	81.88	119.65
PEN3	−0.403	6.345 × 10^−11^	76.12	108.20

## Data Availability

The original contributions presented in this study are included in the article. Further inquiries can be directed to the corresponding author.

## Abbreviations

The following abbreviations are used in this manuscript:

PEEK	Polyetheretherketone
PES	Polyethersulfone
PU	Polyurethane
DCBN	2,6-Dichlorobenzamide
BPA	Bisphenol A
K_2_CO_3_	Potassium carbonate
EP	E-51 epoxy resin
NaCl	Sodium chloride
CYC	Cyclohexanone
NMP	N-methylpyrrolidone
HCl	Hydrochloric acid
